# GII.4 Human Norovirus: Surveying the Antigenic Landscape

**DOI:** 10.3390/v11020177

**Published:** 2019-02-20

**Authors:** Michael L. Mallory, Lisa C. Lindesmith, Rachel L. Graham, Ralph S. Baric

**Affiliations:** Department of Epidemiology, Gillings School of Public Health, University of North Carolina at Chapel Hill, Chapel Hill, NC 27599, USA; mlmallor@live.unc.edu (M.L.M.); lisal@unc.edu (L.C.L.); rlgraham@email.unc.edu (R.L.G.)

**Keywords:** norovirus, antigenic landscape, blockade antibodies, neutralizing antibodies, epitope, antigenic drift, evolution

## Abstract

Human norovirus is the leading cause of viral acute onset gastroenteritis disease burden, with 685 million infections reported annually. Vulnerable populations, such as children under the age of 5 years, the immunocompromised, and the elderly show a need for inducible immunity, as symptomatic dehydration and malnutrition can be lethal. Extensive antigenic diversity between genotypes and within the GII.4 genotype present major challenges for the development of a broadly protective vaccine. Efforts have been devoted to characterizing antibody-binding interactions with dynamic human norovirus viral-like particles, which recognize distinct antigenic sites on the capsid. Neutralizing antibody functions recognizing these sites have been validated in both surrogate (ligand blockade of binding) and in vitro virus propagation systems. In this review, we focus on GII.4 capsid protein epitopes as defined by monoclonal antibody binding. As additional antibody epitopes are defined, antigenic sites emerge on the human norovirus capsid, revealing the antigenic landscape of GII.4 viruses. These data may provide a road map for the design of candidate vaccine immunogens that induce cross-protective immunity and the development of therapeutic antibodies and drugs.

## 1. Review Body

The investigation into human norovirus immunobiology and vaccine development is an essential area of study for the prevention and treatment of viral acute onset gastroenteritis (AGE), particularly in the young, elderly, and immunocompromised. With approximately 20% of all gastroenteritis cases and 200,000 deaths occurring per year primarily due to complications from dehydration and malnutrition, human norovirus-associated infections are a major disease and economic burden, costing 60 billion USD in health care costs and loss of societal productivity globally [1,2,3,4]. As seen with the successful rotavirus vaccine implementation, vaccination programs targeting gastroenteritis have the potential to significantly reduce AGE disease burden [5,6]. Currently, two human norovirus candidate vaccines are in clinical trials. A single-dose GI.I oral adenovirus-based vaccine induced significant effector and memory B cell mucosal immunity [7], while a GI.I/GII.4 vaccine provided protection from homologous virus challenge to GI.1 and decreased symptoms following GII.4 challenge [8,9]. From human challenge studies like these, mucosal IgA, memory IgG, ligand binding blockade Ab, hemagglutination inhibition titer, and serum IgA have been proposed as correlates of human norovirus protective immunity [10,11]. Further, host expression of histo-blood group antigens (HBGAs), specifically secretor positive phenotype, along with pre-existing exposure to older viral strains, play a large role in shaping susceptibility and reaction to emergent GII.4 and other genotype outbreak strains [9,12,13,14]. Vaccine-generated protective immunity typically relies on inducing neutralizing antibodies which mimic natural exposure. Understanding the immunological response to natural and vaccine-induced human norovirus exposure is critical for rational vaccine design.

Belonging to the *Caliciviridae* family, human norovirus is a non-enveloped icosahedral virus, ~40 nm in size, with a ~7.5 kb genome [15]. The single-stranded, positive-sense RNA genome encodes three open reading frames (ORFs), with ORF1 comprising the replicase polyprotein and ORFs 2 and 3 comprising the major (VP1) and minor (VP2) capsid proteins, respectively [16]. When ORF2 is expressed in both baculovirus and Venezuelan Equine Encephalitis (VEE) virus vector systems, monomeric proteins dimerize and 90 copies of the dimer self-assemble into virus-like particles (VLP), which are virtually indistinguishable from native virions [17,18,19]. The capsid monomers can be structurally divided into shell (S) and protruding domains (P). The S domain (residues 1‒221 GII.4 numbering) forms the structural core, while two P domains wrap around each other to form the base unit dimer [20]. Isolated dimers retain select functional features of particles including ligand-binding and some antigenic sites [21,22]. The P domain is further subdivided into P1 (residues 222‒274 and 418‒539) and P2 domains (residues 275‒417) [20,23]. The P1 domain forms a stalk that projects the P domain away from the shell surface, creating space for structural flexibility. The P2 domain forms the most surface-exposed apex of the particle and contains the ligand-binding domain and immunodominant neutralizing antibody epitopes [23,24,25,26].

Currently, there are five classified genogroups of norovirus (GI-V), with genogroups GI and GII causing the majority of human infections. Within GI and GII genogroups there are >30 identified genotypes. GI and GII share less than 50% VP1 identity, with genotypes having less than 20% homology [27,28]. This extensive genetic diversity translates to antigenic variation, which presents a major obstacle to broad-based protective immunity following infection and vaccination. GII strains cause ~90% of outbreaks [29,30], driven primarily by strains within the GII.4 genotype. Recently, the circulation of GII.17 and GII.2 strains temporarily increased during the 2014/2015 and 2016/2017 norovirus seasons, respectively [31,32,33]. However, 50%–70% of yearly human norovirus outbreaks are caused by GII.4 strains, with pandemic levels of infection occurring every ~2–7 years [34,35,36]. The first known human norovirus pandemic occurred 1995–1997, with the causative agent named GII.4 US95/96 [37]. Compared to an endemic GII.4 strain that circulated prior to the pandemic (Camberwell, GII.4 1987), GII.4 US95/96 (named GII.4 1997 in figures) strains are antigenically similar but bind to an expanded number of HBGAs, which are cellular co-factors required for human norovirus infection [25,35,38]. Subsequent pandemics occurred in 2002, 2004, 2006, 2009, and 2012. The persistence of GII.4 strains is directly related to changes in VP1 resulting in altered ligand binding and antigenic drift [35,36,39].

Each subsequent pandemic strain within the GII.4 genotype displays a unique HBGA affinity and antigenicity profile reflective of VP1 mutations, as measured via in vitro assays. Efforts have focused on the binding profiles of the various genotypes of human norovirus to host HBGA, along with monoclonal (mAbs) and polyclonal antibody responses upon both infection and vaccination with VLP. Until recently, an in vitro cell culture system for cultivating human norovirus and modeling virus infection, growth, and antibody neutralization was lacking, limiting the study of the dynamics of virus‒host interaction. Surrogate neutralization assays, or blockade assays, were developed, which measure the ability of antibodies to block human norovirus VLP binding to HBGA-containing substrates, mimicking natural ligand binding [40,41]. Applicable HBGA ligand substrates include human salivary samples (HBGA expression dependent upon the donor), pig gastric mucin (HBGA H, A, and Lewis Y), and synthetic carbohydrate moieties representing different functional groups (for example α1,2-fucose (H antigen) or α1,4-fucose (Lewis antigen)) [42,43]. Antibodies able to block the binding of VLP to these ligand substrates correlate with human norovirus protective immunity [44,45].

The recent development of an in vitro cell culture system utilizing human intestinal enteroid cells (HIE) allows the direct measurement of human norovirus neutralization, although the complexity of the HIE-based system requirements currently limits studies to small sample sizes and contemporary viral strains [46,47]. Supporting epidemiological observations, in vitro virus replication correlated with the secretor phenotype of HIE. Secretor-positive HIE supported GII.4 propagation, while both secretor-positive and -negative HIE supported GII.3 propagation [46]. Importantly, all tested blockade antibody/sera have also neutralized the virus in the HIE culture system, supporting the relevance of the blockade assay as a surrogate neutralization assay [46,48]. Future applications of the human norovirus HIE system and antibody blockade assay may identify additional mechanisms of virus sterilization independent of the inhibition of particle‒ligand interactions, as not all protective antibodies serve neutralizing functions, as seen in some influenza and HIV non-neutralizing antibodies that are protective against infection [49,50].

**Figure 1:**
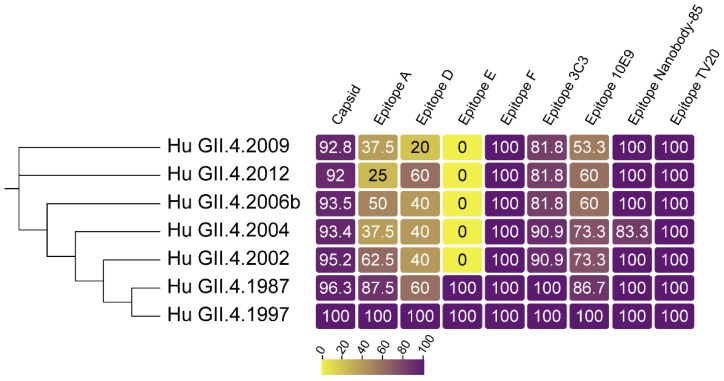
GII.4 VP1 diversity over time. Sequence identity of VP1 (capsid), and known blockade antibody epitopes compared to GII.4 US95/96 (represented by GII.4 1997, AFJ04707.1), the first known GII.4 pandemic strain. GII.4 2002, 2004, 2006b, 2009, and 2012 are sequential pandemic strains, represented by isolates, AAZ31376.2, AFJ04709.1, AEX91909.1, and AFV08794.1, respectively. GII.4 1987 (AAK50355.1) is an endemic GII.4 strain that circulated before GII.4 US95/96 emergence. Overall identity within VP1 is high. Identity within the known evolving blockade antibody epitopes is less well conserved, resulting in the emergence of new pandemic strains refractive to herd immunity shaped by previous GII.4 exposure. Epitopes conserved between GII (epitope nanobody-85) and GI/GII strains (epitope TV20) remain largely unchanged over time in GII.4 strains.

The human norovirus VP1 displays immunodominant antigenic sites. The hypervariable P2 subdomain drives a majority of the blocking antibody response while antibodies to the less variable P1 and shell domains tend to be more cross-reactive and not blocking. Determining the specific residues within the capsid that interact with antibodies has been essential to understanding the antigenic relationship between genotypes and how GII.4 viral evolution escapes herd immunity and drives pandemic outbreaks. Monoclonal antibody-based epitope mapping has identified both blocking and non-blocking epitopes on the GII.4 VP1. Functionally, these epitopes are divided into two categories: those that inhibit VLP binding to the ligand (blockade epitope) and those that do not inhibit VLP from binding to the ligand (non-blocking epitope). Loss of the GII.4 antibody binding/function can be traced to specific capsid amino acid changes within the P2 subdomain of GII.4 VP1 when mapped chronologically (Figure 1) [25,39,51]. Antibody-P domain dimer co-crystal structures further enhance the understanding of these interactions, displaying a 3D representation of antibody binding to specific residues on the P dimer capsid structure [52,53]. Mapping antibodies that bind to conserved regions on multiple viruses pinpoints potential areas on the virus for the preferential targeting of vaccine and drug design and represents a key future area of study.

Ideally, vaccination platforms would induce broadly blocking antibodies to conserved epitopes within GI and GII strains for cross-genogroup protection, demonstrating the importance of mapping these interactions. Broadly cross-reactive epitopes common between GI and GII [54,55,56,57] or within GII strains [58,59] have been identified, primarily occurring within the distal region of the P domain, near the shell/P1 interface or within the shell domain (Table 1). Antibody access to these epitopes is likely restricted by steric hindrance in vivo, as many of these antibodies do not bind intact particles. Nanobodies, small (15‒25 kDa), single-domain camelid immunoglobins comprised of the VH and VL variable domains, binding to these occluded epitopes, indicate that human norovirus particles are capable of significant conformational changes that allow transient access to occluded epitopes, under certain conditions [59]. Monoclonal antibodies to these epitopes have not yet demonstrated blockade or neutralization activity; however, their breadth of reactivity could facilitate other functions for protection from infection including the demarcation of antigen for phagocytosis, as well as reagents for diagnostic assays.

**Figure 2:**
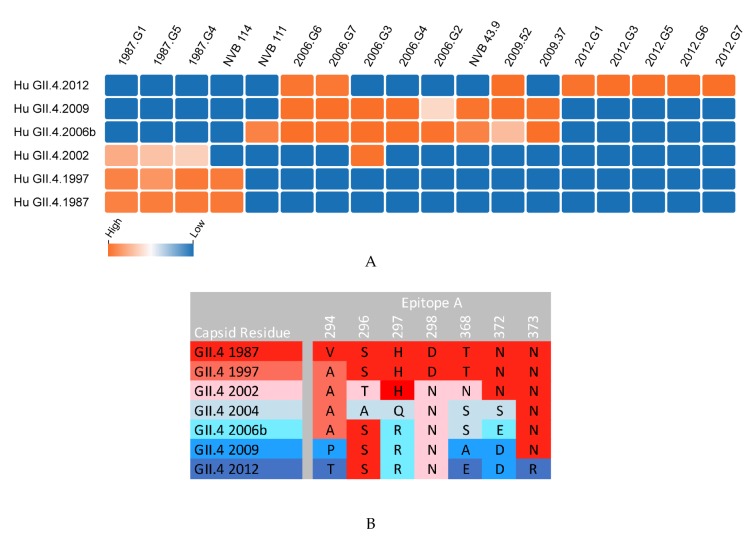
Mouse and human anti-epitope A monoclonal antibody blockade of time-ordered GII.4 strain virus-like particles (VLP). (**A**) Monoclonal antibodies to epitope A were generated in response to the immunization of mice with GII.4 VLPs (1987, 2006, 2009, 2012 mAbs) or the infection of humans (NVB mAbs), and their IC_50_ for the blockade of VLP-ligand binding declined from highly potent (orange) to no inhibition (blue) as reported in References [25,26,38,39,60,61]. GII.4 2004 data not available. (**B**) Antigenic drift within epitope A limits the breadth of mAb recognition of epitope A.

**Figure 3:**
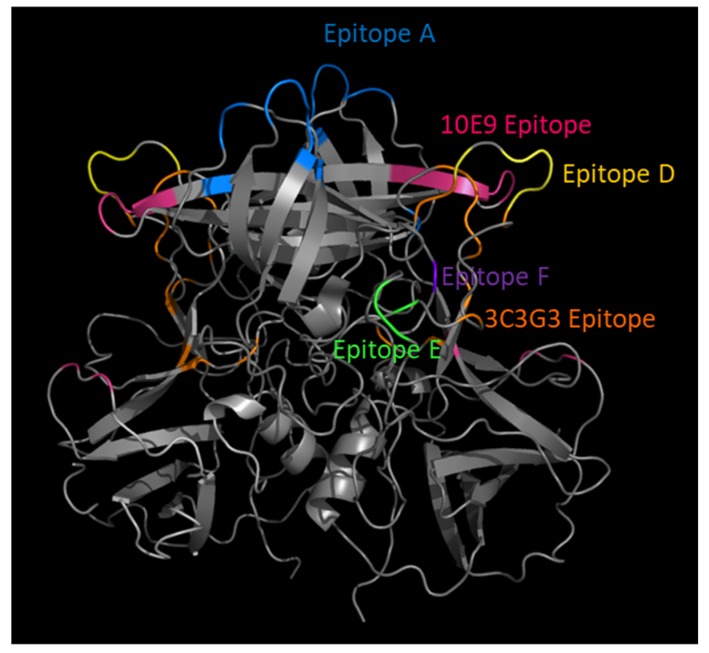
Ligand-blockade antibody epitopes are surface exposed and usually within hypervariable loops within the VP1 P2 subdomain. GII.4 2012 P dimer homology model (4OP7) with blocking antibody epitopes color coded.

Significant effort has been applied to defining GII.4 blockade antibody epitopes and their functions, which has led to the characterization of two general classes of blockade antibody epitopes (Table 1). The first is defined by highly variable, surface-exposed epitopes, while the second class represents epitopes located more distal to the particle apex and subsequently guarded by conformational-restricted antibody access. Falling into the first class, epitope A is a hypervariable, immunodominant epitope comprising ~40% of the serum blockade antibody response [25,26,51,61,62]. Located at the apex of the dimer surface in the P2 subdomain, anti-A mAbs are potent at blocking ligand interactions. mAbs to this area are often sensitive to even minor changes in epitope sequence, resulting from viral evolution [39,60] (Figure 2A,B). Emergent pandemic GII.4 strains correlate with residue changes within epitope A, leading to a loss of mAb response and reduced polyclonal sera blockade potency (Figure 2A) [25,39,51,62]. Epitope D is also within the first class of blockade epitopes, residing proximal to the particle surface and epitope A within the P2 domain [25]. Amino acid residues here form a loop at the rim of the carbohydrate-binding pocket, making antibodies to epitope D potent at blocking ligand interactions (Figure 3) [25,64]. In addition to impacting Ab binding, sequence variation within epitope D also modulates affinity for select HBGA binding, linking evolution in this domain to both host immunity from infection and host susceptibility to infection [35,60,64]. Two additional blockade antibody epitopes overlapping epitope D have been described, supporting the role of these residues in virus neutralization and HBGA ligand affinity [52,67].

Residing within the second class of blockade epitopes are epitopes E and F. Epitope E is less surface exposed than A or D and more distal from the particle surface where antibody access is more limited and regulated by particle conformation (Figure 3) [42,66]. Epitope F is the only known conserved GII.4 blockade epitope, remaining conserved between GII.4 strains spanning 1974‒2015. Epitope F is less surface exposed than epitopes A, D, or E and located in a depression, hence antibody access to epitope F is restricted [25,65,66]. Antibody access to epitopes E and F is temperature-dependent, supporting the hypothesis that human norovirus particles are dynamic structures that explore various conformations that affect epitope exposure, hiding epitopes capable of providing broad cross-protective immunity [65,66]. In addition to temperature, antibody access to epitope F is under allosteric regulation by the NERK motif, including residues 234, 310, 316, 483, 494, which mediates particle conformation [65,66]. Supporting the allosteric effect of the NERK motif and particle dynamics on broadly blocking/neutralizing antibody binding, escape from a broadly neutralizing antibody to mouse norovirus is mediated by a mutation distal to the antibody binding site that likely affects particle conformation [70]. For vaccine design, preferential immunogen presentation targeting the specific conserved anchor residues for neutralizing antibodies has the potential to induce universal protection. It must also be considered that targeting these regions can place evolutionary pressure on the virus to change these epitopes, leading to immune escape variants, and ultimately to novel outbreak/pandemic strains of greater antigenic diversity [71,72].

Although mouse immunization has been the primary source of monoclonal antibody generation and epitope mapping, techniques to develop human monoclonal antibodies following norovirus exposure are currently being applied [25,48]. Human mAbs following infection and vaccination support epitope mapping findings following mouse immunization, reflecting similar binding patterns of known epitopes [25]. All antibodies isolated contribute to our understanding of human norovirus immunobiology, with antibodies generated in naïve mice being reflective of the vaccine’s potential target population—very young children—while antibodies generated in adults with multiple exposure histories may guide cross-protective immunogen design or function as therapeutic or diagnostic antibodies. Importantly, GII.4 norovirus infection and immunization elicit antibodies to cross-GII.4 blocking epitopes that may be exploited for vaccine or drug platforms [25,41].

Extensive research has gone into mapping and analyzing the antibody interactions with specific residues on human norovirus VP1. As more antibodies and their corresponding residues are being identified and validated using both crystallographic and genetics-based epitope exchange techniques, blockade/binding patterns of antibodies begin to emerge that distinguish blocking/neutralizing vs. non-blocking/neutralizing potential and help determine the mechanism of antibody-mediated inhibition of ligand binding for general areas on the viral capsid (Table 1). Potent blockade antibodies typically recognize residues within loop structures where variation does not affect particle integrity (Figure 3). Antibody binding to these epitopes can prevent ligand binding by either direct or indirect steric hindrance of the binding pocket [25,58,67,73]. Antibodies to less-accessible epitopes may block ligand binding by indirect steric hindrance, modulating particle dynamics needed for binding or by inducing particle disassembly [53,65,66]. As additional epitopes are defined, an overlap in binding patterns begins to emerge, defining the larger antigenic sites that are targeted by overlapping antibodies across the antigenic landscape of human norovirus VP1, as described for HIV and influenza [74,75,76]. For example, mAb panels have begun this process for the capsid dimer apex using epitope A mAbs. Key residues within epitope A serve as anchors for different types of epitope A mAbs. Specifically, residues 294, 298, 368, and 373 are essential for specific mAb-binding footprints, indicating that epitope A is an antigenic site comprising multiple epitopes [39,60,61]. Additional antigenic sites are likely at the P1/P2 domain interface (recognized by epitope E, F, and G mAbs and nanobody 85 [42,58,66]) and at the P/Shell domain interface (recognized by B518 mAb [53]), among other unidentified regions.

Defining these antigenic sites will inform rational human norovirus vaccine design. Targeting multiple epitopes within an antigenic site, rather than a single highly neutralizing epitope, will curtail the virus’s ability to rapidly evolve an escape mutation in response to vaccine-induced immunity [76,77,78]. This strategy may be particularly effective when applied to multiple conserved epitopes that require a cost in viral fitness to change, such as residues mediating particle assembly and integrity. Although antibodies to conserved epitopes are rarer than antibodies to variable epitopes, multiple conserved neutralizing antibody epitopes have been described for HIV1, influenza, and human norovirus [65,79,80]. Serological repertoire studies following vaccination and infection will expand the number of available antibodies and facilitate the mapping of additional epitopes [81,82,83]. By the modification of VLP conformation, particle stabilization, or scaffolding techniques, VLP immunogens with an optimal presentation of these epitopes can then be designed, and potentially yield improved cross-protective immunity to human noroviruses.

By implementing state-of-the-art technologies, we are progressing towards eliciting human norovirus protective immunity through vaccination. The study of serological responses after primary and repeat infection and immunization have produced rich panels of antibodies that bind distinct epitopes and have been essential for the mapping of the functional domains of the viral capsid. As more antibody binding patterns and functions are discovered, overlap in binding residues suggests that the broadening of norovirus antigenic epitopes into larger antigenic sites is warranted. Preferential targeting of these antigenic sites in vaccine design could be the key to successful human norovirus cross-protective vaccination platforms. In addition to research efforts, effective public health measures that increase access to and education of vaccination benefits are critical for the success of a vaccine program.

## Figures and Tables

**Table 1:** GII.4 human norovirus antibody epitopes. GII.4 monoclonal antibodies (mAbs) bind to GII.4-specific blockade epitopes located in the P2 subdomain (blue shading), GII cross-reactive epitopes located in the P1/P2 domains (green), or GI/GII cross-reactive regions located primarily in the shell domain (grey) and C-terminal P1 domain. Superscripts: a—GII.10 numbering; b—nanobodies known to induce particle disassembly are not categorized as blockade antibodies in this study.

VP1 Epitope	VP1 Domain	Features	Reference
Epitope A: 294–298, 368, 372, 373	P2	Hypervariable: immunodominant blocking; predictive of new strain	[25,26,39,51,60,61,62]
Epitope D: 391, 393–396	P2	Variable; blocking; regulates HBGA affinity	[24,35,39,63,64]
Epitope E: 407, 412, 413	P2	Variable; Ab access particle conformation-dependent	[42]
Epitope F: 327, 404	P2	Conserved GII.4 1987–2015 blocking; Ab access particle conformation-dependent	[65,66]
3C3G3 Epitope: 245, 247, 389, 390, 397, 435, 443–446, 448	P2/P1	Variable; blocking; Residue 397 modulates HBGA interaction	[67]
10E9 Epitope Chain A: 391, 394, 395, 397, 341, 435, 444, 446, 448, 504, 506; Chain B: 340–343, 345	P2/P1	Blocking and neutralizing; spans both monomers of the dimer	[52]
Nanobody-26 Epitope Chain A: 231, 488. Chain B 269, 271, 272, 274, 276, 316, 470–472, 475 ^a^	P2/P1	GII cross-reactive; spans both monomers of the dimer; nanobody binding induces particle disassembly ^b^	[58]
Nanobody-85 Epitope: 520–522, 524‒526	P1	GII cross-reactive; site occluded on intact particles; nanobody binding induces particle disassembly	[58,59]
5B18 Epitope: 433, 496, 530, 533–535 ^a^	P1	GII cross-reactive; site occluded on intact particles	[53]
NV23, NS22 Epitope: 453–472	P1	GI, GII cross-reactive	[54]
MAB 14-1 Epitope: 418 to 426 and 526 to 534	P1	GI, GII cross-reactive	[68]
1B4, 1f6, 8D8 and 10B11 Epitope: 31–60	Shell	GI, GII cross-reactive; site occluded on intact particles	[55,69]
TV20 Epitope: 52–56	Shell	GI, GII cross-reactive; non-blocking	[56]
N2C3 Epitope: 55–60	Shell	Human and animal norovirus cross-reactive	[57]
